# YOD1 drives acute kidney injury by deubiquitinating Bax and promoting apoptosis in tubular epithelial cells

**DOI:** 10.7150/thno.132208

**Published:** 2026-06-25

**Authors:** Ying Zhao, Shijie Fan, Yu Fang, Qingqing Zhao, Qian Zhou, Zheng Xu, Hong Zhu, Guang Liang

**Affiliations:** 1 Department of Endocrinology, the First Affiliated Hospital of Wenzhou Medical University, Wenzhou, Zhejiang 325035, China.; 2 Chemical Biology Research Center, School of Pharmaceutical Sciences, Wenzhou Medical University, Wenzhou, Zhejiang 325035, China.; 3 Department of Pharmacy and Institute of Inflammation, Zhejiang Provincial People's Hospital, Affiliated People's Hospital, Hangzhou Medical College, Hangzhou, Zhejiang 310014, China.; 4 Zhejiang Provincial Key Laboratory of Drug Discovery and Safety Evaluation for Inflammatory Chronic Diseases, School of Pharmacy, Hangzhou Medical College, Hangzhou 310014, Zhejiang, China.

**Keywords:** acute kidney injury, deubiquitination, YOD1, Bax, apoptosis

## Abstract

**Rationale:**

Acute kidney injury (AKI) is a life-threatening clinical syndrome characterized by high mortality, in which tubular epithelial cell death represents a key pathological event. Emerging evidence underscores the importance of the ubiquitin system in the progression of AKI. Here, we focus on the function of YOD1 in AKI.

**Methods:**

We generated tubular epithelial cells (TECs)-specific *Yod1* knockout mice (YOD1CKO) by crossing *Yod1^fl/fl^* mice and *Ggt1-cre* mice. Both YOD1CKO mice and *Yod1^fl/fl^* littermates were subjected to cisplatin- or ischemia/reperfusion (I/R)-induced AKI models. Through co-immunoprecipitation (Co-IP) combined with LC-MS/MS analysis, we identified potential substrate proteins of YOD1.

**Results:**

We observed that YOD1 is predominantly expressed in TECs and is upregulated during AKI injury. Renal tubular specific *Yod1* knockout significantly alleviated tubular damage and apoptosis in AKI mice. Mechanistically, we identified the pro-apoptotic protein Bax as a direct substrate of YOD1. YOD1 removes K63-linked ubiquitin chains from Bax at lysine 128 via its catalytic cysteine residue C155, thereby promoting Bax activation, and mitochondrial translocation and subsequent apoptosis. YOD1 failed to promote apoptosis in Bax-deficient cells, confirming Bax as the essential downstream mediator.

**Conclusions:**

Our study reveals a previously unrecognized YOD1-Bax regulatory axis that drives tubular apoptosis in AKI, and highlights YOD1 may hold therapeutic potential.

## Introduction

Acute kidney injury (AKI) is a prevalent clinical syndrome characterized by rapid renal function decline, resulting in elevated serum creatinine, reduced glomerular filtration rate, and high morbidity and mortality 1,2. The pathological hallmarks include sublethal and lethal damage to tubular epithelial cells (TECs), typically triggered by ischemia/reperfusion (I/R) injury, nephrotoxic agents, or sepsis 3. Epidemiological studies show that AKI occurs in 2-31% of hospitalized patients, and among critically ill patients, the mortality rate may exceed 50% 4. Recovered patients with AKI also face a higher risk of developing chronic kidney disease and end-stage kidney disease, which highlights the long-term social and economic burden of this disease 5. Although AKI is of great clinical significance, the molecular mechanism behind its pathogenesis is still not fully clear, which limits the development of targeted and effective treatment methods.

Apoptosis of TECs is the core pathological process that drives the progression of AKI and renal function decline 6-8. Their loss directly impairs the reabsorption and secretion function of the kidneys, which leads to a decrease in glomerular filtration rate 9,10. Pharmacological inhibition of apoptotic pathways has demonstrated renal protective effects in AKI models. For example, administration of caspase inhibitors alleviates tubular apoptosis and renal dysfunction in AKI mice 11. Similarly, targeting Bcl-2 family proteins or enhancing anti-apoptotic signals preserves tubular integrity and reduces kidney injury under AKI condition 12,13. These findings show the potential of targeted TECs apoptosis for the treatment of AKI.

Existing evidence shows ubiquitination is an important regulator of cell death and survival pathways 14. Deubiquitinating enzymes (DUBs) have attracted increasing attention for their ability to reverse ubiquitination and regulate key signal events 15. By analyzing the transcriptome public data of cisplatin and ischemic reperfusion-induced AKI mouse models, we found the upregulation of YOD1 in the damaged renal tubules. YOD1 contains an N-terminal ubiquitin-binding domain and an OTU catalytic domain 16. YOD1 was initially characterized as a regulatory molecule of endoplasmic reticulum-related degradation (ERAD). Subsequent studies have found that it participates in a variety of cell processes, including protein quality control, inflammatory signaling and cell fate determination 17,18. YOD1 cleaves both K48-linked and K63-linked ubiquitin chains, which indicates that it has a wide range of substrate specificity and functional diversity 16. However, its expression pattern, biological function, and pathological relevance in the kidney remain unexplored.

Here, we investigate the role of TECs-specific YOD1 in AKI. We show that knockout of *Yod1* in TECs ameliorates cisplatin and I/R-induced kidney injury and suppresses tubular apoptosis. Mechanistically, YOD1 directly interacts with and deubiquitinates BCL-2-associated X protein (Bax), thereby promoting its activation and driving tubular apoptosis. Our findings reveal a YOD1-Bax regulatory axis in AKI and propose YOD1 as a candidate target for treating AKI.

## Materials and Methods

### Antibodies

Primary antibodies against Flag (#20543), HA (#51064), His (#66005-1-IG), and GAPDH (#60004) were supplied by Proteintech (Wuhan, China). Antibody against YOD1 (#A13270) was supplied by ABclonal (Wuhan, China). Antibodies against Bax (#ET1603-34), Bcl-2 (#ET1702-53), Cleaved Caspase-3 (#ET1602-47), Pro Caspase-3 (#ET1602-39), KIM-1(#HA721535), and Cytochrome C (#ET1610-60) were obtained from HUABIO (Hangzhou, China). Antibody against Ubiquitin (#3936) was acquired from Cell Signaling Technology (MA, USA). Antibody against Tom20 (#ab186735) was sourced from Abcam (Cambridge, UK).

### Animal experiments

Tubular epithelial cells (TECs)-specific *Yod1* knockout mice (Ggt1-Cre *Yod1^flox/flox^* mice, YOD1CKO) were generated and genotyped from Model Organisms Center (Shanghai, China) through crossing *Yod1^flox/flox^* mice with *Ggt1*-Cre transgenic mice. All animals were kept in a controlled environment (12 h/12 h light-dark cycle, 22 ± 2 °C, 55 ± 5% humidity) and provided with standard rodent chow and water. All animal procedures received approval from the Animal Policy and Welfare Committee of Wenzhou Medical University (Approval No. Wydw2021-0182). These procedures were carried out in line with the NIH Guide for the Care and Use of Laboratory Animals. Experimental procedures followed ARRIVE guidelines with randomized, blinded design.

(1) Cisplatin-induced acute kidney injury mouse model.

Male YOD1CKO mice (8-12 weeks old, 22-25 g) and *Yod1^flox/flox^* (WT) mice were randomly divided to control or experimental groups (n = 6 per group): WT-Ctrl, WT-cisplatin, YOD1CKO-Ctrl, and YOD1CKO-cisplatin. The cisplatin (25 mg/kg, i.p.) was prepared in 0.9% sterile saline. Equivalent volumes of sterile saline were injected in the control groups 19.

(2) Ischaemia/reperfusion (I/R)-induced acute kidney injury mouse model.

Male YOD1CKO mice (8-12 weeks old, 22-25 g) and *Yod1^flox/flox^* (WT) mice were randomly assigned into to sham or I/R groups. Mice underwent anesthetized with 3% isoflurane, followed by placement on a heating pad to preserve body temperature at 37 °C. Following a midline laparotomy, ischemia was induced by clamping both renal pedicles with microvascular clamps, as evidenced by the kidneys turning from red to dark purple. Reperfusion was allowed by removing the clamps after 28 minutes of ischemia, and the abdominal incision was closed with sutures. The same operation was carried out in sham control mice, without clamping of the renal pedicles 19.

Following 72 hours of cisplatin treatment or I/R surgery, the mice received an intraperitoneal injection of sodium pentobarbital for euthanasia. Blood samples were obtained and centrifuged (3,000 ×g, 10 min) to obtain serum for analysis. Serum creatinine (SCr) and blood urea nitrogen (BUN) levels were quantified through a biochemical analyzer (Beckman, Germany).

### Histological analysis

Kidney tissues underwent fixation with 4% polyformaldehyde. Sections of 5 µm thickness were cut with a microtome (Leica, Germany). Hematoxylin & Eosin (H&E) staining was performed to evaluate overall kidney structure. Periodic Acid-Schiff (PAS) staining was used to show the basal membrane and glycogen deposition of renal tubules. The injury score was defined as: 0 (none), 1 (<10%), 2 (10-25%), 3 (26-50%), 4 (51-75%), and 5 (>75%) 20.

### Single-cell RNA sequencing (scRNA-seq)

The scRNA-seq was performed using the 10X Genomics Chromium Single-Cell 3' Kit (v3). Briefly, renal tissues were dissociated into a single-cell suspension using an appropriate dissociation solution. The suspension was then loaded onto a Chromium chip to generate single-cell gel beads-in-emulsion (GEMs). The resulting libraries were sequenced on an Illumina NovaSeq 6000 platform (paired-end, 150 bp) at a minimum depth of 20,000 reads per nucleus at LC-Bio Technology Co., Ltd. Raw data were processed using Cell Ranger (v3.1.0) and aligned to the GRCm38 reference genome. Quality control metrics were as follows: cells with 500-5,000 genes and <25% mitochondrial reads were retained. Downstream analysis was performed using Seurat (v3.1.1) with PCA (top 10 PCs) for dimensionality reduction and t-SNE for visualization. Clusters were identified using the SNN graph-based method, and marker genes were selected using the Wilcoxon rank-sum test (expressed in >10% of cells, logFC > 0.25).

### Immunofluorescence

For immunofluorescence staining, 5 μm-thick frozen kidney tissue sections were fixed in methanol for 10 min. Once fixation was complete, the sections were incubated for 15 min with 0.3% Triton X-100 (T434386, Aladdin, Shanghai, China) in PBS. To reduce nonspecific binding, the samples were then blocked for one hour using 5% bovine serum albumin (BSA, B2064, Sigma-Aldrich, USA). Sections were incubated with primary antibody overnight, then with fluorophore-conjugated secondary antibody for 1 hour. After thorough PBS washes to remove unbound antibodies, the sections were counterstained with DAPI and visualized using a confocal fluorescence microscope.

### Cell culture and transfection

The TCMK1 was sourced from Fuheng Biotechnology Co., Ltd.‌‌ (Shanghai, China). The mouse embryonic fibroblast cell line (NIH/3T3) was acquired from the Cell Bank of Chinese Academy of Sciences (Shanghai, China). TCMK1 cells were maintained in Dulbecco's Modified Eagle Medium (DMEM, Gibco, USA), and NIH/3T3 cells were cultured in RPMI-1640 medium (Gibco, USA). Both types of media received the same supplements: 10% fetal bovine serum (FBS, Gibco, USA) and 1% penicillin-streptomycin (100 U/mL penicillin and 100 μg/mL streptomycin, Sbjbio, China). All cells were kept under standard culture conditions (37 °C, 5% CO₂).

(1) Plasmid construction and transfection.

The mouse expression plasmids encoding Flag-tagged YOD1 (wild-type and cysteine mutants: C155A, H262A, H337A) and His-tagged Bax were generated by Tsingke Biotechnology (Beijing, China). Ubiquitin constructs (HA-tagged wild-type Ub, K63-linked Ub, and K48-linked Ub) were obtained from Addgene. Following the manufacturer's protocol, all plasmids were transiently transfected using Lipofectamine 3000 (L3000150). A mixture ratio of 1:2 (μg plasmid to μl reagent) was employed.

(2) Gene Silencing by small interfering RNA (siRNA) Transfection.

Targeted gene knockdown was achieved using the following siRNA sequences (synthesized by GenePharma, Shanghai). YOD1: 5′- GGGCAAUCGAGAUCUCAAU -3′; Bax: 5′- GUCCUGCUGAGUUGGACUU-3′. Cells were transfected with siRNA using Lipofectamine 2000 (11668500, Thermo Fisher Scientific, USA), following the manufacturer's protocol.

### Real-time quantitative polymerase chain reaction (RT-qPCR)

TRIzol reagent (9109, Takara, Japan) was used to extract total RNA from either kidney tissues or cells. The extracted RNA was then reverse transcribed into cDNA using the HiScript III All in One RT SuperMix Reagent Kit (R333-01, Vazyme, Nanjing, China). For PCR amplification, we applied ChamQ Universal SYBR qPCR Master Mix (Vazyme, Q711-02) on a QuantStudioTM Real-Time PCR detection System. The relative quantification was calculated using the 2^-ΔΔCt^ method.

### Western blot

Protein extraction from kidney tissues or cells carried out with RIPA lysis buffer (BOSTER, Wuhan, China). Equal protein loads were subjected to SDS-PAGE (10% or 12% gel) for separation, followed by transfer to PVDF membranes (10600023, millipore, MA, USA). Membranes were treated with 5% BSA in TBST, kept with the primary antibody overnight. Then, we used HRP-conjugated secondary antibody to incubate the membranes at room temperature for 1 hour. Target proteins were visualized through enhanced chemiluminescence (ECL, P10300, NCM Biotech, Suzhou, China). ImageJ software (version 1.38e, NIH, Bethesda, MD) was used to quantify the intensity of the band.

### Co-immunoprecipitation (Co-IP) and LC-MS/MS analysis

For Co-IP, cells or tissues were lysed in NP-40 buffer (P0013F, Beyotime, Shanghai, China). The lysate was incubated with primary antibody or control IgG overnight. Then add protein A/G beads to capture the immune complex for 4 hours. The beads were washed 5 times with lysis buffer. They were then mixed again in loading buffer and boiled for Western blot analysis. The specific protein interaction was confirmed by analyzing the Input samples, IP samples, and IgG controls.

The LC-MS/MS analysis was completed by Aimsmass (Shanghai, China). In short, we prepared NIH/3T3 cell samples of transfected empty Flag carrier or Flag-YOD1 plasmid. These samples were injected into the liquid chromatography system combined with the tandem mass spectrometer. Polypeptides or metabolites were separated by reverse phase chromatography. The separated substance was ionized by electric spray ionization. The obtained ions were analyzed in the tandem mass spectrometry mode to obtain the fragment spectrum. These spectra were used to identify and quantify target compounds by matching against a database or standard curves.

### TUNEL staining

The One Step TUNEL Apoptosis Assay Kit (C1089, Beyotime, Shanghai, China) was used to detect cell apoptosis. This method labels fragmented DNA with Cy3-dUTP through Terminal Deoxynucleotidyl Transferase (TdT). Briefly, kidney tissue sections were fixed in 4% paraformaldehyde for 30 min at room temperature, treated with 0.3% Triton X-100. After that, the sample was incubated with the TUNEL reaction mixture at 37 °C. TUNEL-positive cells showing red fluorescence were quantified using fluorescence microscopy (Nikon, Japan).

### Mitochondrial membrane potential (MMP) determination

MMP was measured using the fluorescent probe JC-1 (HY-K0601, MCE, New Jersey, USA). The cells cultured in a 6-well plate were incubated with JC-1 (2 μM) at 37 °C for 20 minutes. MMP was evaluated under a fluorescence microscopy. JC-1 shows a change from red fluorescence (aggregates, indicating high MMP) to green fluorescence (monomers, reflecting MMP loss).

### Isolation of subcellular fractions

The Cell Mitochondria Isolation Kit (C3601, Beyotime, Shanghai, China) was used to separate cytoplasmic and mitochondrial fractions from cells. Briefly, 20-50×10^6 TCMK1 cells were homogenized in 1-2.5 ml mitochondria isolation buffer and incubated on ice for 15 min. Sequential differential centrifugation was then performed: initial clarification at 600×g for 10 min to remove nuclei and cell debris, followed by 11,000 ×g centrifugation for 10 min to pellet the mitochondrial fraction. The resulting pellet contained purified mitochondria, while the supernatant represented the cytosolic fraction devoid of mitochondrial components.

### Flow cytometry

An Annexin V FITC/PI Apoptosis Detection Kit (556547, BD Biosciences, NJ, USA) was used to determine the extent of cell apoptosis. Briefly, 1×10^5 cells were placed into 100 μL of 1× Binding Buffer, stained with 5 μL Annexin V-FITC for 15 min and 5 μL PI for 5 min in the dark. Apoptosis was immediately analyzed using a CytoFLEX flow cytometer (Beckman Coulter, CA, USA), and data were processed with FlowJo software.

### Statistical analysis

Data are reported as mean ± SD based on at least three independent runs of each experiment. GraphPad Prism 8.0 served to conduct the statistical analyses. Normality and variance homogeneity were tested. We used two tailed Student's t test for comparisons between two groups. For multiple group comparisons, one-way or two- way ANOVA was applied, followed by Bonferroni's post hoc test. P value less than 0.05 were considered significant (* P < 0.05, ** P < 0.01, *** P < 0.001).

## Results

### YOD1 expression is upregulated in AKI and exacerbates tubular epithelial cell injury

**Figure 1:**
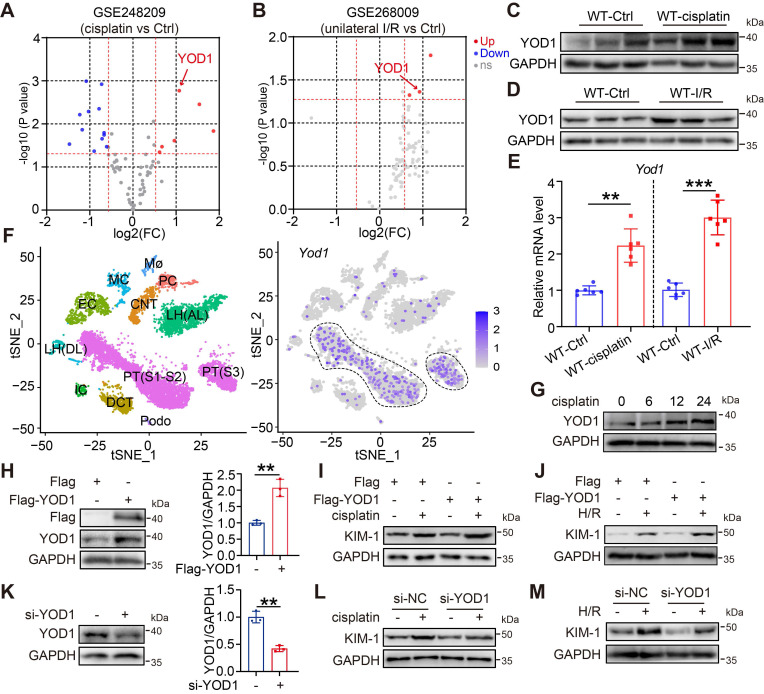
** YOD1 expression is upregulated in AKI and exacerbates tubular epithelial cell injury.** (A) The mRNA profile of DUBs in cisplatin-induced AKI mice was derived from published transcriptome data (GSE248209). (B) The mRNA profile of DUBs in unilateral I/R-induced AKI mice was showed from published transcriptome data (GSE268009). (C-E) Western blotting and RT-qPCR analysis showing YOD1 expression levels for kidney tissue of WT and AKI mice (n = 6). (F) Identification of main cell types in wild-type mouse kidney by tSNE (left). Distribution of *Yod1* expression (purple) among each cell type (right). (G) Western blot analysis showing YOD1 expression in TCMK1 cells in response to cisplatin at various time points (n = 3). (H) Western blot analysis showing Flag expression in YOD1-overexpressing TCMK1 cells (n = 3). (I-J) Flag-YOD1-expressing TCMK1 cells were stimulated with cisplatin or H/R for 12 hours. Representative immunoblot of KIM-1 expression (n = 3). (K) Representative western blot of YOD1 expression in TCMK1 cells transfected with si-YOD1 sequences (n = 3). (L-M) TCMK1 cells transfected with si-YOD1 were stimulated with cisplatin or H/R for 12h. Representative immunoblot of KIM-1 expression (n = 3).

To explore the potential function of DUBs in AKI, we analyzed publicly available Gene Expression Omnibus (GEO) datasets (GSE248209 for cisplatin-induced AKI and GSE268009 for I/R-induced AKI). Among all DUBs, the gene expression of YOD1 was consistently upregulated in both AKI models (Figure 1A-B, Figure S1A-B). We further validated this finding *in vivo* using mouse models of AKI, where Western blot analysis revealed a marked increase in YOD1 protein levels within injured kidney tissues compared with the sham controls (Figure 1C-D, Figure S1C-D). Consistent with these results, quantitative PCR revealed a corresponding elevation in YOD1 mRNA levels during AKI (Figure 1E), suggest that YOD1 upregulation occurs primarily at the transcriptional level. To determine the cellular localization of YOD1 expression in the kidney, we assessed scRNA-seq data from mouse kidneys. Expression profiling revealed that YOD1 is mainly expressed in tubular epithelial cells (TECs), with minimal expression in other cell types (Figure 1F). Immunohistochemical staining also confirmed that YOD1 is located in renal tubular epithelial cells and has a stronger signal in the kidneys of AKI mice (Figure S1E). Moreover, YOD1 expression in TCMK1 cells was induced by cisplatin treatment in a time-dependent manner (Figure 1G, Figure S1F).

We next explored the functional role of YOD1 in TECs by transfecting TCMK1 cells with a Flag-tagged YOD1 overexpression plasmid (Figure 1H). Upon exposure to cisplatin or hypoxia/reoxygenation (H/R) to mimic AKI *in vitro*, YOD1 overexpression markedly exacerbated tubular cell injury, as shown by a rise in the protein levels of the injury marker kidney injury molecule 1 (KIM-1) (Figure 1I-J, Figure S1G-H). Conversely, siRNA-mediated knockdown of YOD1 attenuated the cisplatin- or H/R-induced upregulation of KIM-1 (Figure 1K-M, Figure S1I-J), suggesting a protective effect against tubular cell injury. Overall, these findings demonstrate that YOD1 in TECs acts as a driving factor in injured kidneys.

### TECs-specific *Yod1* knockout attenuates cisplatin-induced renal tubule injury and apoptosis

**Figure 2:**
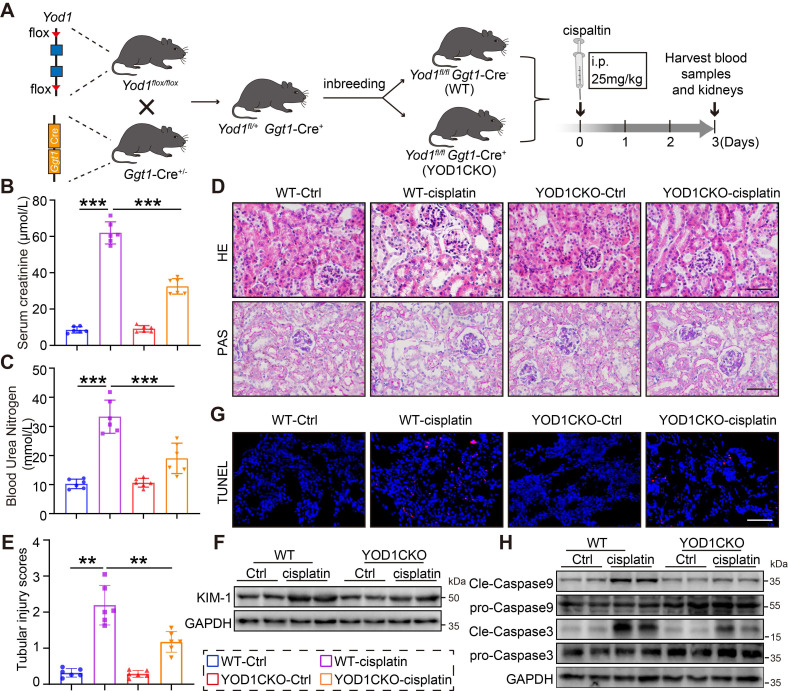
** TECs-specific *Yod1* knockout attenuates cisplatin-induced renal tubule injury and apoptosis.** (A) Generation of TECs-specific *Yod1* knockout mice (YOD1CKO) and establishment of cisplatin-induced AKI mouse model. (B-C) Measurements of Scr and BUN in mice (n = 6). (D) Histological assessment of mouse kidneys using H&E and PAS. Scale bar: 50 μm. (E) Sections were evaluated to assign tubular injury scores for assessing renal damage (n = 6). (F) Western blot analysis showing KIM-1 expression levels in the kidneys of each group (n = 6). (G) Representative images of TUNEL staining in mice. Scale bar: 50 μm. (H) Western blot analysis showing Cleaved-Caspase9 and Cleaved-Caspase3 expression levels in the kidneys (n = 6).

We obtained TECs-specific *Yod1* knockout mice (YOD1CKO) through breeding of *Yod1^flox/ flox^* mice with *Ggt1*-Cre mice (Figure 2A). Genotyping of the YOD1CKO mice was confirmed by tail DNA PCR (Figure S2A). The TECs-specific knockout of *Yod1* did not affect normal development, general health, or survival of the mice. Both YOD1CKO mice and *Yod1^flox/ flox^* (WT) littermates were subjected to cisplatin-induced nephrotoxicity via intraperitoneal injection to establish an AKI model (Figure 2A). Following cisplatin challenge, YOD1CKO mice showed lower levels of SCr and BUN compared to WT littermates (Figure 2B-C). H&E and PAS staining further revealed attenuated renal damage in YOD1CKO mice, with less tubular dilation and better preservation of brush border integrity (Figure 2D). These morphological improvements were corroborated by a significant decrease in semiquantitative tubular injury scores (Figure 2E). Consistent with the structural improvements, expression of KIM-1 was significantly lower in YOD1CKO kidneys after cisplatin exposure (Figure 2F, Figure S2B). Furthermore, TUNEL staining showed fewer apoptotic cells in YOD1CKO renal sections compared to WT (Figure 2G). Additionally, YOD1 knockout significantly reduced cleavage of caspase9 and caspase3 but had no effect on caspase8 (Figure 2H, Figure S2D), indicating that YOD1 primarily regulates the intrinsic mitochondrial apoptotic pathway. Collectively, these findings indicate that specific *Yod1* knockout in TECs protects against cisplatin-induced renal injury.

### TECs-specific *Yod1* knockout attenuates I/R-induced renal tubule injury and apoptosis

**Figure 3:**
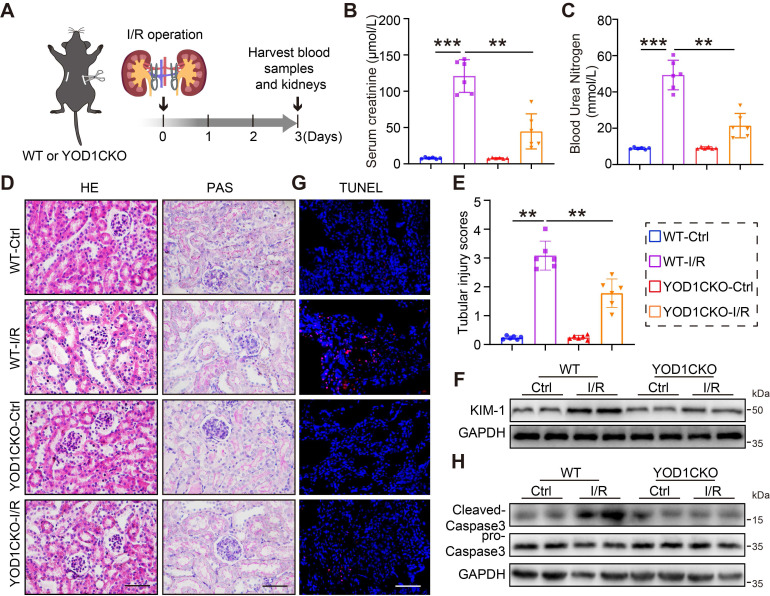
** TECs-specific *Yod1* knockout attenuates I/R-induced renal tubule injury and apoptosis**. (A) The establishment of I/R-induced AKI mouse model. (B-C) Measurements of Scr and BUN in mice (n = 6). (D) Histological assessment of mouse kidneys using H&E and PAS. Scale bar: 50 μm. (E) Sections were evaluated to assign tubular injury scores for assessing renal damage (n = 6). (F) Western blot analysis showing KIM-1 expression levels in the kidneys of each group (n = 6). (G) Representative images of TUNEL staining in mice. Scale bar: 50 μm. (H) Western blot analysis showing Cleaved-Caspase3 expression levels in the kidneys (n = 6).

We next examined the role of YOD1 deletion in renal I/R injury, another major model of AKI. WT and YOD1CKO mice received bilateral renal ischemia for 28 minutes or sham surgery, followed by 3 days of reperfusion (Figure 3A). I/R-challenged YOD1CKO mice exhibited significantly attenuated of renal dysfunction, as shown by lower levels of SCr and BUN than WT littermates (Figure 3B-C). Histopathological analysis further revealed reduced renal damage in YOD1CKO mice, characterized by diminished tubular injury (Figure 3D). Consistent with these findings, semiquantitative tubular injury scores and expression of KIM-1 were both significantly lower in I/R-challenged YOD1CKO mice (Figure 3E-F, Figure S3A). TUNEL staining combined with Western blot analysis confirmed that YOD1CKO attenuates I/R-induced renal apoptosis (Figure 3G-H, Figure S3B). These results suggest that YOD1CKO also confers protection against I/R-induced AKI in mice.

### YOD1 physically interacts with the pro-apoptotic protein Bax in TECs

**Figure 4:**
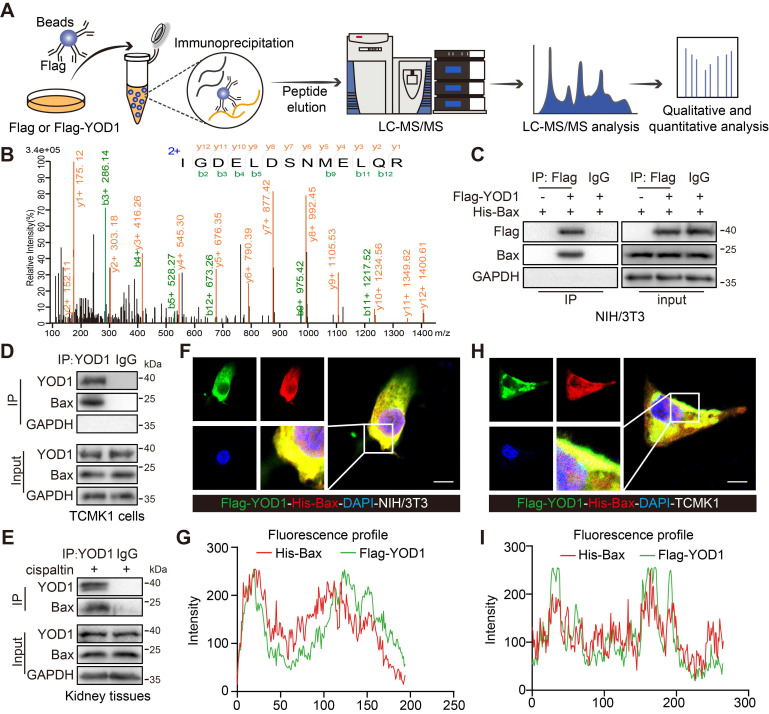
** YOD1 physically interacts with the pro-apoptotic protein Bax in TECs.** (A) Flag or Flag-YOD1 plasmids went into NIH/3T3 cells. Cell lysates were mixed with anti-Flag and protein A/G beads for co-immunoprecipitation. Bound proteins were eluted, fragmented into peptides, and analyzed by LC-MS/MS. (B) The peptide IGDELDSNMELQR (derived from Bax) as seen on LC-MS/MS. (C) NIH/3T3 cells were transfected with Flag-YOD1 and His-Bax plasmids. Exogenous YOD1 was immunoprecipitated using anti-Flag antibody (n = 3). (D-E) Immunoprecipitation of endogenous YOD1 from TCMK1 cells and kidney tissues (n = 3). (F-G) Colocalization of Flag-YOD1 (green) with His-Bax (red) in NIH/3T3 cells were assessed by immunofluorescence and their fluorescence intensity profiles. Scale bar, 5 μm. (H-I) Immunofluorescence colocalization of Flag-YOD1 (green) with His-Bax (red) in TCMK1 cells and their fluorescence intensity profiles. Scale bar, 5 μm.

To elucidate the molecular mechanism by which YOD1 mediates tubular cell injury and apoptosis, we sought to identify its direct protein substrates. As a deubiquitinating enzyme (DUB), YOD1 typically functions by binding to and modulating specific target proteins. We performed an unbiased protein analysis via LC-MS/MS in YOD1-overexpressing cells to identify potential binding partners (Figure 4A). Among the candidates, the pro-apoptotic regulator Bax emerged as a potential substrate of YOD1 in TECs (Figure 4B, Figure S4A-B). Given the critical role of Bax in mitochondrial-dependent apoptosis during AKI 21, we prioritized its validation. Co-immunoprecipitation (co-IP) assays in NIH/3T3 cells co-transfected with Flag-YOD1 and His-Bax confirmed the exogenous interaction (Figure 4C). Furthermore, we found the endogenous interaction between YOD1 and Bax in both cisplatin-stimulated TCMK1 cells and kidney tissues (Figure 4D-E). Immunofluorescence and colocalization analyses further demonstrated substantial colocalization of YOD1 with Bax in both NIH/3T3 and TCMK1 cells (Figure 4F-I). Moreover, confocal microscopy revealed that YOD1 is predominantly cytoplasmic with partial mitochondrial colocalization (Figure S4C), and upon cisplatin stimulation, Bax translocated from the cytoplasm to mitochondria as evidenced by increased colocalization with the mitochondrial marker (Figure S4D). Collectively, these results demonstrate a direct interaction between YOD1 and Bax, and support the functional relevance of Bax mitochondrial translocation in TECs during AKI.

### YOD1 regulates the K63-linked deubiquitination of Bax at residue K128 via its active site C155

**Figure 5:**
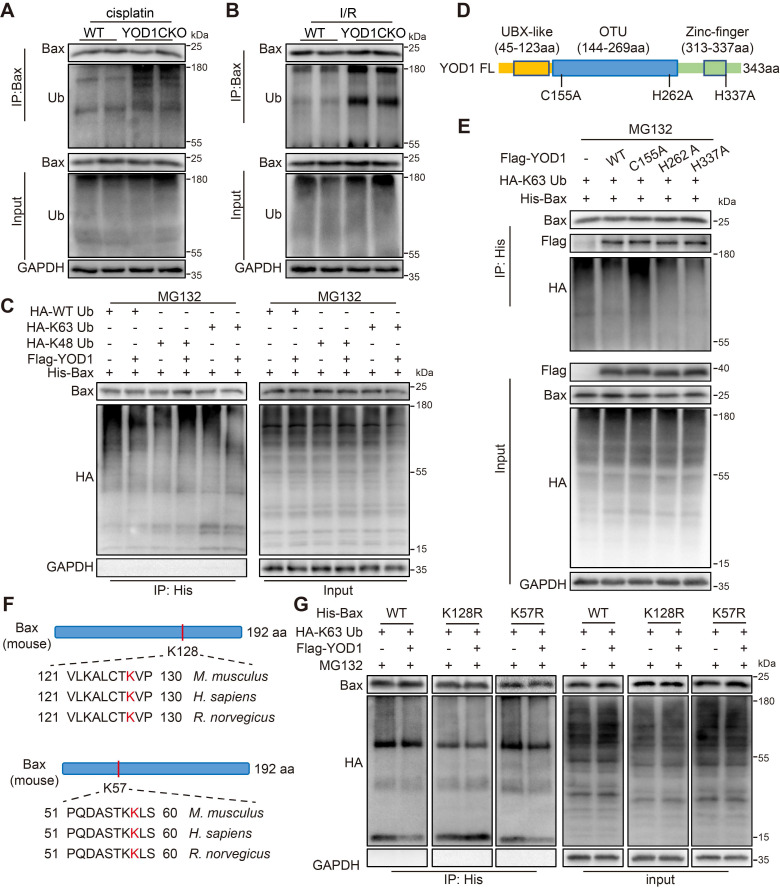
** YOD1 regulates the K63-linked deubiquitination of Bax at residue K128 via its active site C155.** (A-B) Ubiquitination of Bax was detected by immunoblotting using anti-Ubiquitin antibody in the kidneys (n = 3). (C) NIH/3T3 cells were co-transfected with Flag-YOD1 plasmid, His-Bax plasmid and HA-Ub, K48 or K63 plasmids. Twenty-four hours after transfection, cells were treated with MG132 (10 μM) for 6 hours before lysis. Ubiquitinated Bax was detected by immunoblotting using anti-HA antibody (n = 3). (D) Schematic of the YOD1 active-site variant (C155A, H262A, H337A) construct. (E) His-Bax and HA-K63 plasmids were transfected into NIH/3T3 together with Flag-YOD1 (WT, C155A, H262A, H337A) and then subjected to 10 μM MG132 for 6 hours. Ubiquitinated Bax was detected by immunoblotting using an anti-HA antibody (n = 3). (F) Schematic illustration of the Bax ubiquitinated-lysine residue (K128 and K57) mutation construct. (G) Transfections into NIH/3T3 included His-Bax (WT, K128R or K57R), HA-K63, and Flag-YOD1. MG132 (10 μM) was added for 6 hours. Anti-HA antibody detected ubiquitinated Bax by western blotting (n = 3).

We next investigated whether YOD1 regulates Bax through direct deubiquitination. Ubiquitination assays revealed that Bax ubiquitination was significantly enhanced in renal tissues of YOD1CKO mice subjected to either cisplatin- or I/R-induced AKI (Figure 5A-B). In contrast, overexpression of YOD1 in TCMK1 cells reduced Bax ubiquitination (Figure S5A). Additionally, YOD1 overexpression did not alter Bax Ser184 phosphorylation or total acetylation levels (Figure S5B), confirming that YOD1 specifically regulates Bax via deubiquitination. To define the linkage specificity, we utilized HA-tagged ubiquitin mutants with only K48 or K63, the two most typical sites involved in ubiquitin chain formation. Co-IP analyses showed that YOD1 removed K63-linked polyubiquitin chains from Bax, with no significant effect on K48-linked ubiquitination (Figure 5C). Based on sequence homology, we then mutated three conserved putative catalytic residues of YOD1 (C155, H262, H337) to alanine 22 (Figure 5D). Notably, the C155A mutation abolished YOD1-mediated removal of K63-linked ubiquitin chains from Bax, while mutations in H262 or H337 showed no effect (Figure 5E), indicating C155 as the essential catalytic residue for YOD1 deubiquitinating Bax.

Finally, to identify the ubiquitination site on Bax specifically targeted by YOD1, we used an online E3 ubiquitin ligase site prediction tool and identified two evolutionarily conserved lysine residues, K57 and K128, in Bax protein (Figure 5F, Figure S5C). Subsequent assays showed that mutation of K128 to arginine (K128R) markedly reduced basal ubiquitination of Bax and abolished further deubiquitination by YOD1. In contrast, the K57R mutation showed no effect (Figure 5G). Together, these results demonstrate that YOD1 directly removes K63-linked ubiquitin chains from Bax at lysine 128 via its catalytic cysteine residue C155.

### YOD1 limits Bax-Bcl-2 interaction and drives mitochondrial apoptosis in TECs

**Figure 6:**
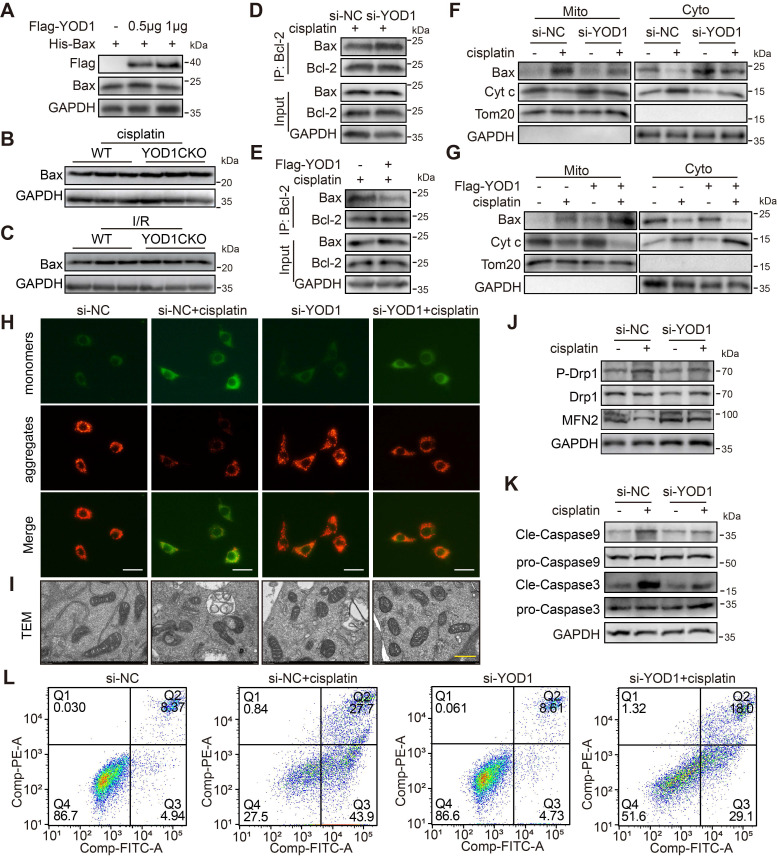
** YOD1 limits Bax-Bcl-2 interaction and drives mitochondrial apoptosis in TECs.** (A) Western blot analysis showing Bax expression levels in TCMK1 cells under a Flag-YOD1 dose gradient (n = 3). (B-C) Representative immunoblot of Bax from kidney tissues (n = 6). (D-E) NIH/3T3 cells transfected with si-YOD1 or Flag-YOD1 were subjected to Co-IP with an anti-Bcl-2 antibody (n = 3). (F-G) After transfection with si-YOD1 or Flag-YOD1, TCMK1 cells were exposed to cisplatin for 8 hours. Western blot analysis of Bax and Cyt *c* protein expression in mitochondria (Mito) and cytoplasm (Cyto) (n = 3). (H) TCMK1 cells transfected with si-YOD1 were stimulated with cisplatin for 12 hours. Representative images of JC-1 staining on the mitochondrial transmembrane. Scale bar, 25 μm. (I) TCMK1 cells transfected with si-YOD1 were stimulated with cisplatin for 24 hours. Representative TEM images showing mitochondrial ultrastructure in indicated groups. Scale bar, 500 nm. (J-K) After transfection with si-YOD1, TCMK1 cells were exposed to cisplatin for 24 hours. Western blot analysis showing protein levels of P-Drp1, MFN2, Cleaved-Caspase9 and Cleaved-Caspase3 (n = 3). (L) After transfection with si-YOD1, TCMK1 cells were exposed to cisplatin for 36 hours. Cell apoptosis was quantified using Annexin V/PI.

We then examined whether YOD1 influences the stability or activity of Bax protein in TECs. Our data demonstrated that neither overexpression nor knockout of YOD1 affected Bax protein levels both *in vitro* and *in vivo* (Figure 6A-C), thereby excluding the possibility that YOD1 regulates Bax protein stability. We therefore hypothesized that YOD1 might regulates Bax activity. A key regulatory step in Bax activation is its dissociation from the inhibitory partner Bcl-2 23. Co-IP assays revealed that YOD1 knockdown enhanced the interaction between Bax and Bcl-2, whereas YOD1 overexpression weakened this association (Figure 6D-E). These findings suggest that YOD1 limits Bax-Bcl-2 interaction and promotes the release of active Bax from its restrained state (Bax/Bcl-2 complex).

We next examined the downstream mitochondrial events associated with Bax activation. Cisplatin-induced Cytochrome c (Cyt c) release and Bax mitochondrial translocation were markedly suppressed by YOD1 knockdown (Figure 6F), but enhanced by YOD1 overexpression in TECs (Figure 6G). Consequently, YOD1 knockdown preserved the cisplatin-induced loss of mitochondrial membrane potential (MMP), as indicated by a reduced JC-1 aggregate/monomer ratio (Figure 6H), while overexpression exacerbated the MMP in cisplatin-challenged TECs (Figure S6A). Transmission electron microscopy (TEM) revealed that YOD1 knockdown alleviated cisplatin-induced mitochondrial ultrastructural damage, including cristae disruption and matrix swelling, whereas YOD1 overexpression aggravated these morphological abnormalities (Figure 6I and Figure S6B). Furthermore, Western blot analysis demonstrated that YOD1 knockdown increased the expression of the mitochondrial fusion protein MFN2 and decreased the level of the fission marker p-Drp1 (Ser616), while YOD1 overexpression exerted opposite effects (Figure 6J and Figure S6C). Finally, both Western blot assessment of apoptotic markers and Annexin V/PI staining by flow cytometry confirmed that YOD1 knockdown protected TECs from cisplatin-induced apoptosis, whereas YOD1 overexpression sensitized cells to the apoptotic challenge (Figure 6K-L, Figure S6D-J). In summary, our findings indicate that YOD1 does not affect Bax protein stability but enhances its pro-apoptotic function by disrupting the Bax/Bcl-2 complex formation.

### YOD1 promotes tubular apoptosis via deubiquitinating Bax in TECs

**Figure 7:**
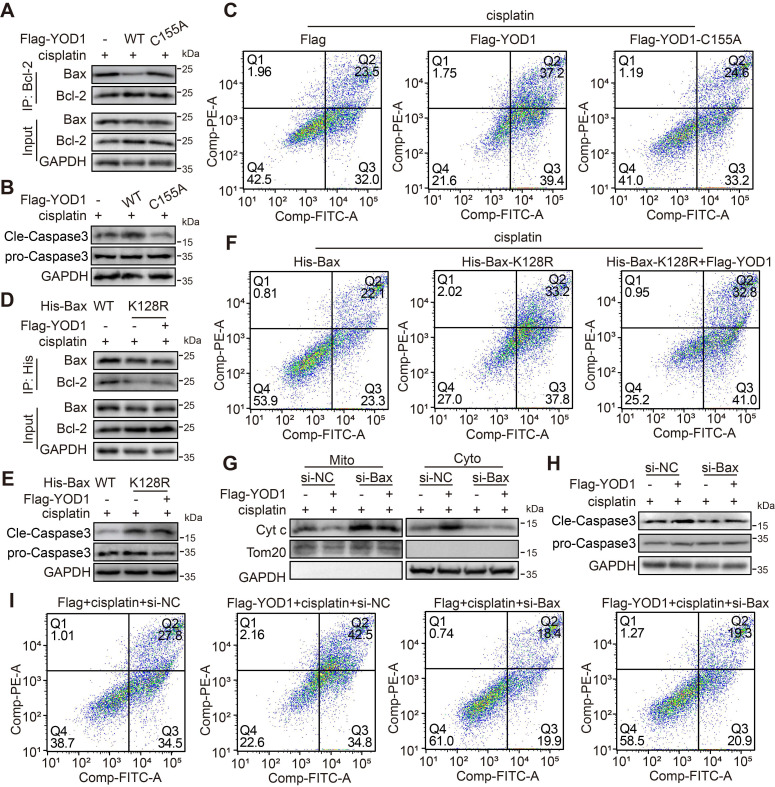
** YOD1 promotes tubular apoptosis via deubiquitinating Bax in TECs.** (A) TCMK1 cells expressing Flag-YOD1 (WT or C155A) was subjected to Co-IP with an anti-Bcl-2 antibody (n = 3). (B-C) Flag-YOD1 (WT or C155A) was transfected into TCMK1 cells and then stimulated by cisplatin for 36 hours. Representative western blot analysis showing protein levels of Cleaved-Caspase. Cell apoptosis levels were measured using Annexin V/PI staining (n = 3). (D) After transfection of His-Bax (WT or K128R) into TCMK1 cells (with or without Flag-YOD1), lysates were immunoprecipitated with an anti-His antibody (n = 3). (E-F) His-Bax (WT or K128R) and Flag-YOD1 were transfected into TCMK1 cells and then stimulated by cisplatin for 36 hours. Representative western blot analysis showing protein levels of Cleaved-Caspase. Annexin V/PI staining assay was performed to detect the level of cell apoptosis (n = 3). (G) TCMK1 cells received Flag-YOD1 and si-Bax , then were exposed to cisplatin for 8 hours. Representative immunoblot of Bax and Cyt *c* protein expression in mitochondria (Mito) and cytoplasm (Cyto) (n = 3). (H-I) TCMK1 cells received Flag-YOD1 and si-Bax , then were exposed to cisplatin for 36 hours. Representative western blot analysis showing protein levels of Cleaved-Caspase. Cell apoptosis was quantified using Annexin V/PI (n = 3).

To elucidate whether YOD1-induced Bax activation and cell apoptosis depends on Bax deubiquitination, we performed function analyses using catalytic and substrate mutants. First, we confirmed that the deubiquitinase activity of YOD1 is indispensable for this process. Under cisplatin treatment, overexpression of the catalytically inactive mutant YOD1-C155A failed to disrupt the Bax-Bcl-2 interaction, in contrast to wild-type YOD1 (Figure 7A). Consequently, YOD1-C155A did not promote mitochondrial depolarization (Figure S7A), caspase-3 cleavage, or apoptosis in TECs (Figure 7B-C, Figure S7B-C), indicating that the pro-apoptotic function of YOD1 strictly relies on its deubiquitinating activity. We next investigated whether Bax deubiquitination at K128 serves as the critical step in this cascade. Cells expressing the ubiquitination-deficient mutant Bax-K128R exhibited reduced binding to Bcl-2 (Figure 7D), accompanied by enhanced caspase-3 activation and increased apoptosis (Figure 7E-F, Figure S7D-E). Notably, overexpression of YOD1 did not further enhance apoptosis in Bax-K128R-expressing cells, suggesting that Bax deubiquitination at K128 is necessary for YOD1-driven TECs apoptosis.

To further test the functional dependency of YOD1 on Bax, we knocked down Bax using siRNA in TECs (Figure S7F). Depletion of Bax abolished the apoptotic response to cisplatin, as shown by suppressed Cytochrome c release, diminished caspase-3 cleavage, and reduced apoptosis (Figure 7G-I, Figure S7G). Under this condition, YOD1 overexpression failed to promote TECs apoptosis, confirming that Bax is the essential downstream effector through which YOD1 exerts the pro-apoptotic function. In summary, these findings demonstrate that YOD1 catalyzes deubiquitination of Bax at K128, leading to disruption of the inhibitory Bax/Bcl-2 complex and consequent unleashing of Bax-mediated apoptosis in TECs.

## Discussion

In this work, we establish that the deubiquitinating enzyme YOD1 is upregulated in TECs during AKI and promotes tubular apoptosis through directly targeting Bax. Through comprehensive analyses, we demonstrate that YOD1 removes K63-linked ubiquitin chains from Bax at lysine 128 via its catalytic residue C155, leading to Bax activation and tubular apoptosis. Genetic deletion of *Yod1* in TECs conferred significant protection against renal injury, identifying the YOD1-Bax axis as an important pathogenic pathway and potential therapeutic target in AKI.

Renal tubular epithelial cells have high metabolic activity and play a central role in the pathogenesis of AKI. These characteristics make them vulnerable to damage from low blood flow, toxins and inflammation 24. Apoptosis of renal tubular epithelial cells is a major form of programmed cell death in AKI, leading to loss of tubular structure, decreased reabsorption function and damage to the filtration barrier 6. Clinical evidence from renal biopsy samples of patients who died of septic shock confirms the presence of apoptotic cells in proximal and distal tubules 25. Animal studies also support the renal tubular apoptosis has an important role in AKI 8, and inhibition of this process has been shown to attenuate renal injury and improve kidney function in many experimental models 26,27. In addition, the degree of tubular apoptosis correlates with clinical outcomes in AKI patients 28. Our identification of YOD1 as a key molecular regulator of TECs apoptosis and reveals a potential therapeutic intervention node.

In recent years, DUBs-mediated ubiquitination modifications have received increasing attention in AKI research. More and more DUBs have been confirmed to regulate renal tubular injury through different mechanisms. For example, inhibition of USP14 attenuates hypoxia/reoxygenation-induced cell death, inflammation, and oxidative stress in human renal tubular epithelial (HK-2) cells 29. Upregulation of USP10 mitigates AKI by reducing pyroptosis and inflammatory response in TECs 30,31, whereas USP13 alleviates mitochondrial dysfunction and kidney injury by stabilizing MCL-1 32. According to previous work, JOSD2 exerts a protective effect on TECs by removing ubiquitin from SIRT7, which restrains the SIRT7-NF-κB inflammatory pathway 33. These findings together illustrate the mechanistic diversity by which different DUBs the damage response of TECs in AKI. Our study identifies YOD1 as a new member of this growing list of DUBs. The scRNA-seq and Immunofluorescence analyses confirm that YOD1 is mainly localized in TECs, and its expression increases after injury. This expression pattern highlights the specific association between YOD1 and AKI renal tubular pathological changes, which further suggests its potential as a target for AKI treatment.

Bax is a key molecule in the mitochondrial apoptosis pathway, which drives the development of AKI by mediating renal tubular cell death 34,35. Under physiological conditions, Bax remains inactive in the cytosol through forming a complex with Bcl-2 23. Upon apoptotic signal occurs, the Bax/Bcl-2 complex is disrupted, leading to Bax conformational activation, thus transpositioning to mitochondria, promoting the release of cytochrome c, causing caspase activation and cell apoptosis 36,37. Given this central role, Bax has emerged as a potential therapeutic target. Several pharmacological agents, such as the Bax-inhibiting peptide (BIP), resveratrol, and trimetazidine, have been proven to have a protective effect on the kidneys. These drugs work by changing the expression level or activity of Bax 38-41. However, therapeutic targeting of Bax remains challenging due to the transient and dynamic nature of its activation, and the risk of off-target effects. These questions show that it is necessary to better understand the upstream mechanism that regulates Bax activity. Among them, post-translational modifications such as ubiquitination are worthy of attention, which may provide more accurate treatment strategies.

At present, the understanding of Bax ubiquitinization mainly focuses on its regulation of protein stability. E3 ubiquitin ligases, such as Parkin and TRIM17, target Bax for its proteasome degradation, thereby inhibiting the pro-apoptotic ability of Bax 42,43. However, emerging evidence suggests that ubiquitination can also modulate Bax function independent of degradation. For instance, USP12 regulates Bax by removing K63-linked ubiquitin chains, influencing the function of Bax without altering protein stability in HeLa cells 44. Extending these observations, we identify YOD1 can directly binds to Bax and promote its activation in TECs. Our mechanism studies demonstrate that YOD1 catalyzes the removal of K63-linked ubiquitin chains of Bax at residue K128 via its catalytic site C155. This site-specific deubiquitinization serves as a key regulatory switch. It changes the conformation of Bax and how it binds to other proteins, but does not trigger the degradation of Bax. Based on this molecular mapping, we further explain how site-specific deubiquitination translates into functional regulation during kidney injury. We provide compelling evidence that YOD1-mediated deubiquitination enhances Bax activation by disrupting its inhibitory interaction with Bcl-2. Co-immunoprecipitation assays confirm that YOD1 overexpression significantly weakens the Bax-Bcl-2 interaction, thereby promoting the mitochondrial translocation of free Bax. Crucially, this effect requires both YOD1’s catalytic residue C155 and Bax K128, as perturbation of either component abolishes complex disruption. These findings establish YOD1 as a critical regulator of Bax-Bcl-2 interaction in renal tubular cells and provide unprecedented insights into the residue-specific mechanism by which Bax deubiquitination controls mitochondrial apoptosis in AKI.

Even with these advances, some limits of our work need to be noted. First, although we identified Bax as a critical substrate of YOD1, it is plausible that YOD1 may regulate additional proteins involved in AKI pathogenesis. Future proteomic studies could help uncover other relevant substrates and pathways. Second, there are no specific small-molecule inhibitors available for YOD1, which limits the feasibility of pharmacological intervention experiments in the present study. Nevertheless, our findings that YOD1 is upregulated in injured tubules and that genetic deletion of Yod1 in TECs confers significant protection against AKI highlight YOD1 as a promising therapeutic target. The development of selective YOD1 inhibitors would be an important avenue for future investigation. In conclusion, our study elucidates a novel YOD1-Bax pathway that drives tubular apoptosis in AKI. These findings not only deepen our understanding on post-translational regulation in kidney injury but also identify YOD1 as a promising therapeutic target for AKI.

## Supplementary Material

Supplementary figures and table.

## Data Availability

All data are included within the article or Supplementary Information or available from the authors on request.

## References

[1] Star RA (1998). Treatment of acute renal failure. Kidney Int.

[2] Briggs DL (2020). Disparities in Health Outcomes with Dialysis in the United States Vary by Race. Clin J Am Soc Nephrol.

[3] Yang C, Xu H, Yang D, Xie Y, Xiong M, Fan Y (2023). A renal YY1-KIM1-DR5 axis regulates the progression of acute kidney injury. Nat Commun.

[4] Duann P, Lianos EA, Ma J, Lin PH (2016). Autophagy, Innate Immunity and Tissue Repair in Acute Kidney Injury. Int J Mol Sci.

[5] Hoste EAJ, Kellum JA, Selby NM, Zarbock A, Palevsky PM, Bagshaw SM (2018). Global epidemiology and outcomes of acute kidney injury. Nat Rev Nephrol.

[6] Yu SM, Bonventre JV (2020). Acute kidney injury and maladaptive tubular repair leading to renal fibrosis. Curr Opin Nephrol Hypertens.

[7] Belavgeni A, Meyer C, Stumpf J, Hugo C, Linkermann A (2020). Ferroptosis and Necroptosis in the Kidney. Cell Chem Biol.

[8] Maremonti F, Meyer C, Linkermann A (2022). Mechanisms and Models of Kidney Tubular Necrosis and Nephron Loss. J Am Soc Nephrol.

[9] Lin Q, Li S, Jiang N, Jin H, Shao X, Zhu X (2021). Inhibiting NLRP3 inflammasome attenuates apoptosis in contrast-induced acute kidney injury through the upregulation of HIF1A and BNIP3-mediated mitophagy. Autophagy.

[10] Basnakian AG, Ueda N, Kaushal GP, Mikhailova MV, Shah SV (2002). DNase I-like endonuclease in rat kidney cortex that is activated during ischemia/reperfusion injury. J Am Soc Nephrol.

[11] Kaushal GP, Kaushal V, Hong X, Shah SV (2001). Role and regulation of activation of caspases in cisplatin-induced injury to renal tubular epithelial cells. Kidney Int.

[12] Chien CT, Shyue SK, Lai MK (2007). Bcl-xL augmentation potentially reduces ischemia/reperfusion induced proximal and distal tubular apoptosis and autophagy. Transplantation.

[13] Li JY, Sun XA, Wang X, Yang NH, Xie HY, Guo HJ (2024). PGAM5 exacerbates acute renal injury by initiating mitochondria-dependent apoptosis by facilitating mitochondrial cytochrome c release. Acta Pharmacol Sin.

[14] Guo C, Dong G, Liang X, Dong Z (2019). Epigenetic regulation in AKI and kidney repair: mechanisms and therapeutic implications. Nat Rev Nephrol.

[15] Clague MJ, Urbe S, Komander D (2019). Breaking the chains: deubiquitylating enzyme specificity begets function. Nat Rev Mol Cell Biol.

[16] Ernst R, Mueller B, Ploegh HL, Schlieker C (2009). The otubain YOD1 is a deubiquitinating enzyme that associates with p97 to facilitate protein dislocation from the ER. Mol Cell.

[17] Sun J, Chen F, She L, Zeng Y, Tang H, Ye B (2025). YOD1 regulates microglial homeostasis by deubiquitinating MYH9 to promote the pathogenesis of Alzheimer's disease. Acta Pharm Sin B.

[18] Ye B, Lin W, Jiang Y, Zheng Z, Jin Y, Xu D (2025). Cardiomyocyte-derived YOD1 promotes pathological cardiac hypertrophy by deubiquitinating and stabilizing STAT3. Sci Adv.

[19] Doke T, Mukherjee S, Mukhi D, Dhillon P, Abedini A, Davis JG (2023). NAD(+) precursor supplementation prevents mtRNA/RIG-I-dependent inflammation during kidney injury. Nat Metab.

[20] Doke T, Abedini A, Aldridge DL, Yang YW, Park J, Hernandez CM (2022). Single-cell analysis identifies the interaction of altered renal tubules with basophils orchestrating kidney fibrosis. Nat Immunol.

[21] Singh R, Letai A, Sarosiek K (2019). Regulation of apoptosis in health and disease: the balancing act of BCL-2 family proteins. Nat Rev Mol Cell Biol.

[22] Mevissen TE, Hospenthal MK, Geurink PP, Elliott PR, Akutsu M, Arnaudo N (2013). OTU deubiquitinases reveal mechanisms of linkage specificity and enable ubiquitin chain restriction analysis. Cell.

[23] Suzuki M, Youle RJ, Tjandra N (2000). Structure of Bax: coregulation of dimer formation and intracellular localization. Cell.

[24] Li ZL, Huang MM, Yu MY, Nie DF, Fu SL, Di JJ (2024). Mitochondrial fumarate promotes ischemia/reperfusion-induced tubular injury. Acta Physiol (Oxf).

[25] Lerolle N, Nochy D, Guerot E, Bruneval P, Fagon JY, Diehl JL (2010). Histopathology of septic shock induced acute kidney injury: apoptosis and leukocytic infiltration. Intensive Care Med.

[26] Li B, Xia Y, Mei S, Ye Z, Song B, Yan X (2023). Histone H3K27 methyltransferase EZH2 regulates apoptotic and inflammatory responses in sepsis-induced AKI. Theranostics.

[27] Tian Z, Wu Y, Yi B, Li L, Liu Y, Zhang H (2025). ESCRT III-mediated lysosomal repair improve renal tubular cell injury in cisplatin-induced AKI. Autophagy.

[28] Ostermann M, Lumlertgul N, Jeong R, See E, Joannidis M, James M (2025). Acute kidney injury. Lancet.

[29] Pan J, Zhao J, Feng L, Xu X, He Z, Liang W (2023). Inhibition of USP14 Suppresses ROS-dependent Ferroptosis and Alleviates Renal Ischemia/Reperfusion Injury. Cell Biochem Biophys.

[30] Han B, Zheng Q, Li H, Wang Y, Zhang D (2025). PRDM16 suppresses pyroptosis to attenuate the progression of AKI caused by rhabdomyolysis via upregulation of USP10. Cell Mol Life Sci.

[31] Zhao Q, Zhang R, Wang Y, Li T, Xue J, Chen Z (2024). FOXQ1, deubiquitinated by USP10, alleviates sepsis-induced acute kidney injury by targeting the CREB5/NF-kappaB signaling axis. Biochim Biophys Acta Mol Basis Dis.

[32] Wang Q, Cao S, Sun Z, Zhu W, Sun L, Li Y (2025). USP13 inhibition exacerbates mitochondrial dysfunction and acute kidney injury by acting on MCL-1. Biochim Biophys Acta Mol Basis Dis.

[33] Zhao Y, Zhao QQ, Fan SJ, Xu DY, Lin LM, Luo W (2025). JOSD2 alleviates acute kidney injury through deubiquitinating SIRT7 and negativity regulating SIRT7-NF-kappaB inflammatory pathway in renal tubular epithelial cells. Acta Pharmacol Sin.

[34] Li J, Sun X, Yang N, Ni J, Xie H, Guo H (2023). Phosphoglycerate mutase 5 initiates inflammation in acute kidney injury by triggering mitochondrial DNA release by dephosphorylating the pro-apoptotic protein Bax. Kidney Int.

[35] Garner TP, Reyna DE, Priyadarshi A, Chen HC, Li S, Wu Y (2016). An Autoinhibited Dimeric Form of BAX Regulates the BAX Activation Pathway. Mol Cell.

[36] Hantusch A, Das KK, Garcia-Saez AJ, Brunner T, Rehm M (2018). Bax retrotranslocation potentiates Bcl-x(L)'s antiapoptotic activity and is essential for switch-like transitions between MOMP competency and resistance. Cell Death Dis.

[37] Gupta S, Knowlton AA (2005). HSP60, Bax, apoptosis and the heart. J Cell Mol Med.

[38] Li Y, Yokota T, Gama V, Yoshida T, Gomez JA, Ishikawa K (2007). Bax-inhibiting peptide protects cells from polyglutamine toxicity caused by Ku70 acetylation. Cell Death Differ.

[39] Huang C, Jiang S, Gao S, Wang Y, Cai X, Fang J (2022). Sirtuins: Research advances on the therapeutic role in acute kidney injury. Phytomedicine.

[40] Abdelrahman RS, Abdelsalam RA, Zaghloul MS (2022). Beneficial effect of trimetazidine on folic acid-induced acute kidney injury in mice: Role of HIF-1alpha/HO-1. J Biochem Mol Toxicol.

[41] Amini N, Sarkaki A, Dianat M, Mard SA, Ahangarpour A, Badavi M (2019). The renoprotective effects of naringin and trimetazidine on renal ischemia/reperfusion injury in rats through inhibition of apoptosis and downregulation of micoRNA-10a. Biomed Pharmacother.

[42] Shen J, Yang H, Qiao X, Chen Y, Zheng L, Lin J (2023). The E3 ubiquitin ligase TRIM17 promotes gastric cancer survival and progression via controlling BAX stability and antagonizing apoptosis. Cell Death Differ.

[43] Johnson BN, Berger AK, Cortese GP, Lavoie MJ (2012). The ubiquitin E3 ligase parkin regulates the proapoptotic function of Bax. Proc Natl Acad Sci U S A.

[44] Choi HS, Lim ES, Baek KH (2022). Deubiquitinating Enzyme USP12 Regulates the Pro-Apoptosis Protein Bax. Int J Mol Sci.

