# Supplementary material for: YOD1 drives acute kidney injury by deubiquitinating Bax and promoting apoptosis in tubular epithelial cells
Source: Theranostics. 2026 Jun 25;16(14):7765–79. doi: 10.7150/thno.132208 (PMC13440280; doi:10.7150/thno.132208)
Supplement: Supplementary file 1 [file thnov16p7765s1.pdf]

Supplementary Information

YOD1 drives acute kidney injury by deubiquitinating Bax and promoting mitochondrial apoptosis in tubular epithelial cells

The supplementary file includes 1 Table and 7 Figures.

Supplementary Table 1. Primer sequences for RT-qPCR assay.

Gene	Species	Sequence
β-actin-F	Mouse	CTACCTCATGAAGATCCTGACC
β-actin-R	Mouse	CACAGCTTCTCTTTGATGTCAC
Yod1-F	Mouse	AAGTCTCCCTGTGTGTTTCTTGTCG
Yod1-R	Mouse	ACTCAGAAGGCAGAGGCAGAAGG
Primer1-F	Mouse	ACCAATTTTTCGTTTTCCCTGTGT
Primer1-R	Mouse	GCTCCGCGCTGCATTTCAACTTA
Primer2-F	Mouse	CAGTTCTGTGACCCCCTTCC
Primer2-R	Mouse	CGGCAAACGGACAGAAGCATT

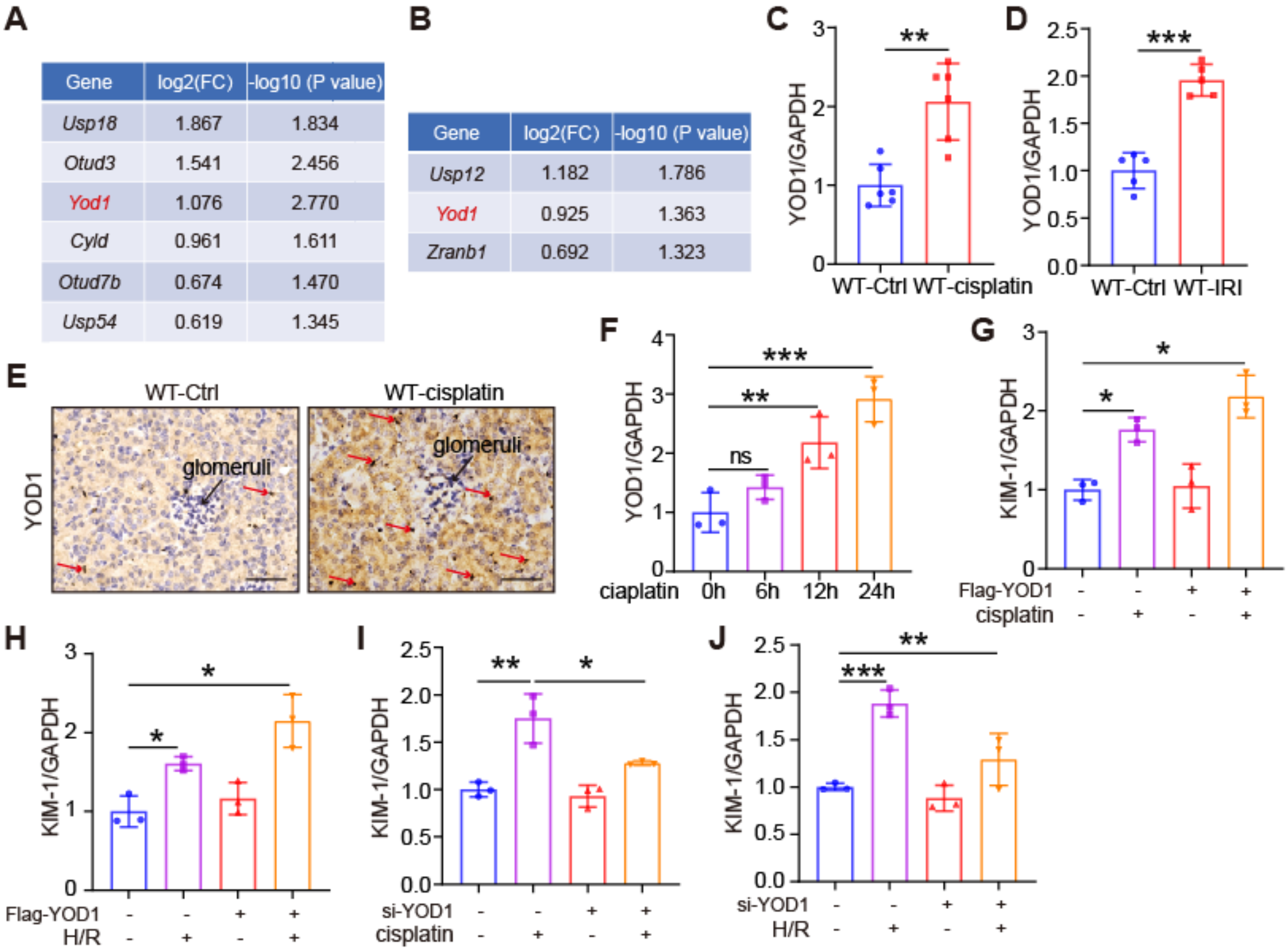

A

Gene	log2(FC)	-log10 (P value)
Usp18	1.867	1.834
Otud3	1.541	2.456
Yod1	1.076	2.770
Cyld	0.961	1.611
Otud7b	0.674	1.470
Usp54	0.619	1.345

B

Gene	log2(FC)	-log10 (P value)
Usp12	1.182	1.786
Yod1	0.925	1.363
Zranb1	0.692	1.323

Supplementary Figure 1

(A-B) The tables show the significantly upregulated DUBs in published transcriptome data (GSE248209 and GSE268009). (C-D) Densitometric quantification of immunoblots in Figure 1C and 1D (n = 6). (E) Representative immunohistochemistry (IHC) images of YOD1 expression in mice. Scale bar, 50 μm. (F) Densitometric quantification of the immunoblot in Figure 1G (n = 3). (G-J) Densitometric quantification of the immunoblots in Figure 1I, 1J, 1L and 1M (n = 3).

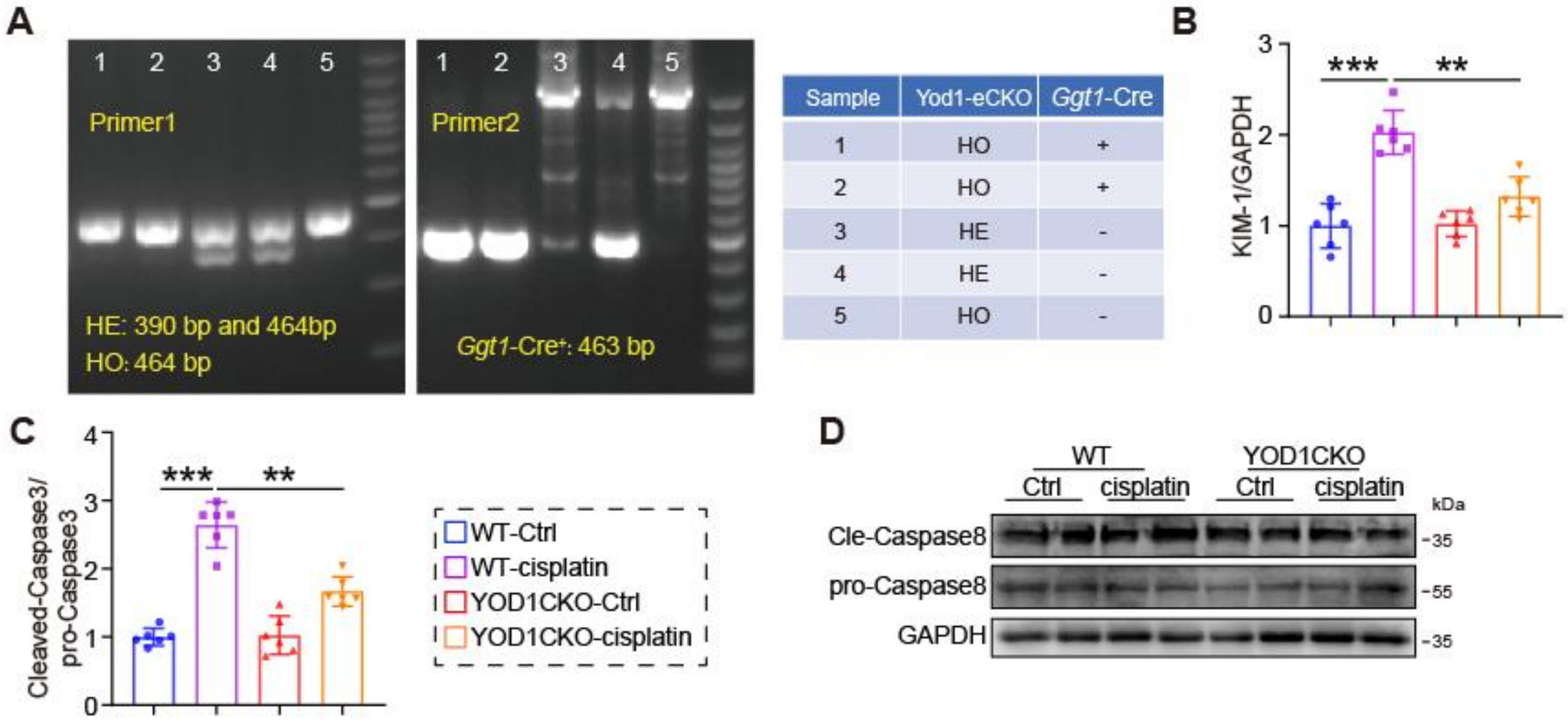

Supplementary Figure 2

(A) The primers of *Yod1* (Primer1, WT:390bp, HO:464bp) and *Ggt1*-Cre (Primer2, 463bp) were respectively used for PCR to identify the genotype of mice. (B-C) Densitometric quantification of the immunoblots in Figure 2F and 2H (n = 6). (D) Western blot analysis showing Cleaved-Caspase8 expression levels of each group (n = 6).

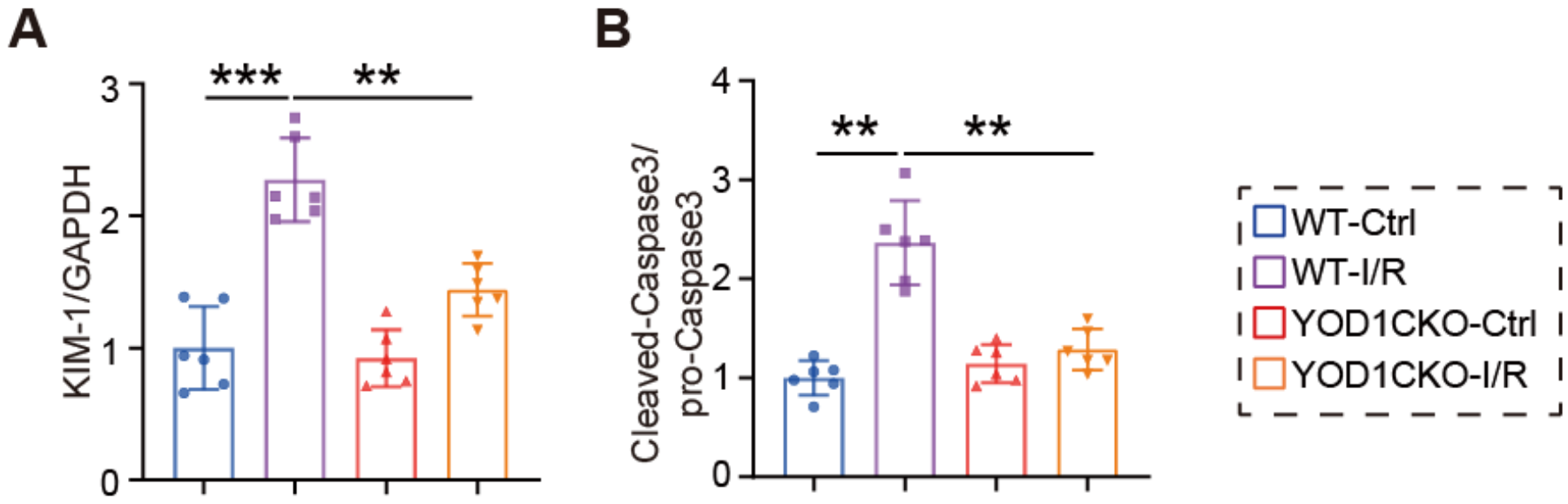

Supplementary Figure 3

(A-B) Densitometric quantification of the immunoblots in Figure 3F and 3H (n = 6).

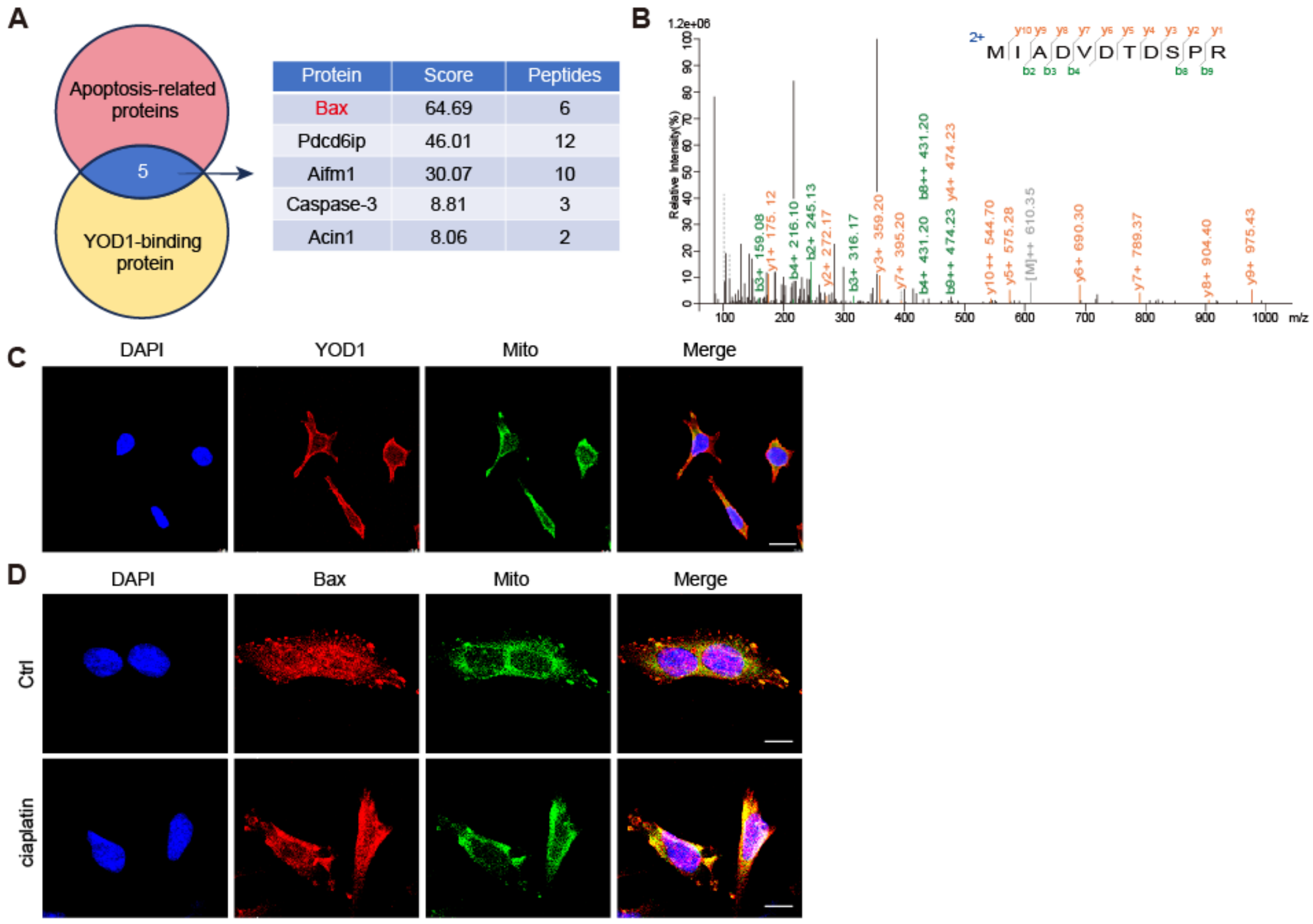

Supplementary Figure 4

(A) Venn diagram showing the overlap between YOD1-interacting proteins and apoptosis-related proteins. (B) The peptide MIADVDTDSPR (derived from Bax) as seen on LC-MS/MS. (C) Colocalization of YOD1 (red) with Mito-Tracker (green) in TCMK1 cells were assessed by immunofluorescence. Scale bar, 25 μm. (D) Colocalization of Bax (red) with Mito-Tracker (green) in TCMK1 cells were assessed by immunofluorescence. Scale bar, 10 μm.

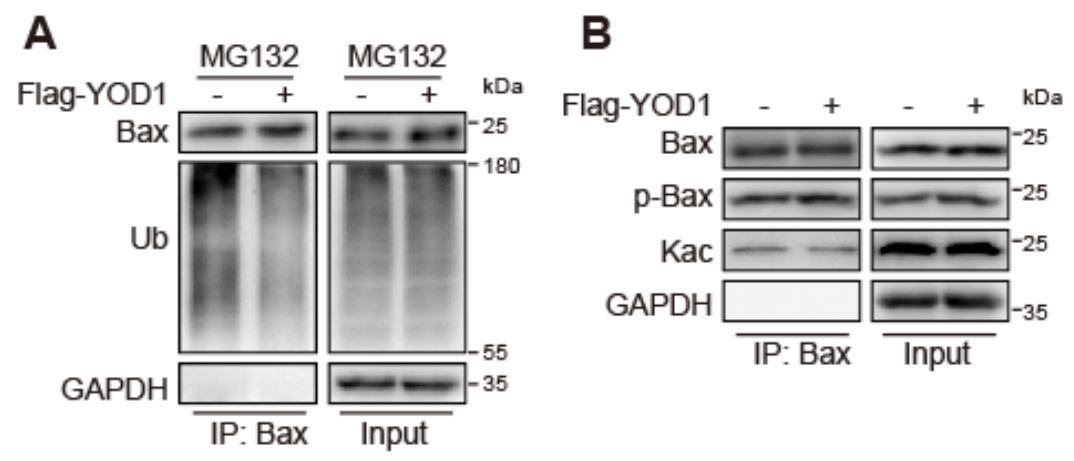

C

General E3 enzymes ubiquitination prediction for Bax (GPS-Uber)					
Protrin	Position	Code	E3 enzymes	Modified sequence	Score
Bax	128	K	General	VLKALCTKVPELIRTIMGWT	0.6981
Bax	57	K	General	PQDASTKKLSECLRRIGDEL	0.2924
Bax	123	K	General	LFYFASKLVLKALCTKVPELI	0.2173

Supplementary Figure 5

(A) Flag-YOD1 was transfected into NIH/3T3 cells and subjected to 10μM MG132 for 6h. An anti-ubiquitin antibody on immunoblots revealed ubiquitinated Bax (n = 3). (B) Immunoprecipitation of endogenous Bax was performed, followed by western blotting for Flag, Bax, p-Bax (Ser184), and acetylated-lysine (Kac) (n = 3). (C) General E3 enzymes ubiquitination prediction for Bax. The website of GPS-Uber is http://gpsuber.biocuckoo.cn/index.php.

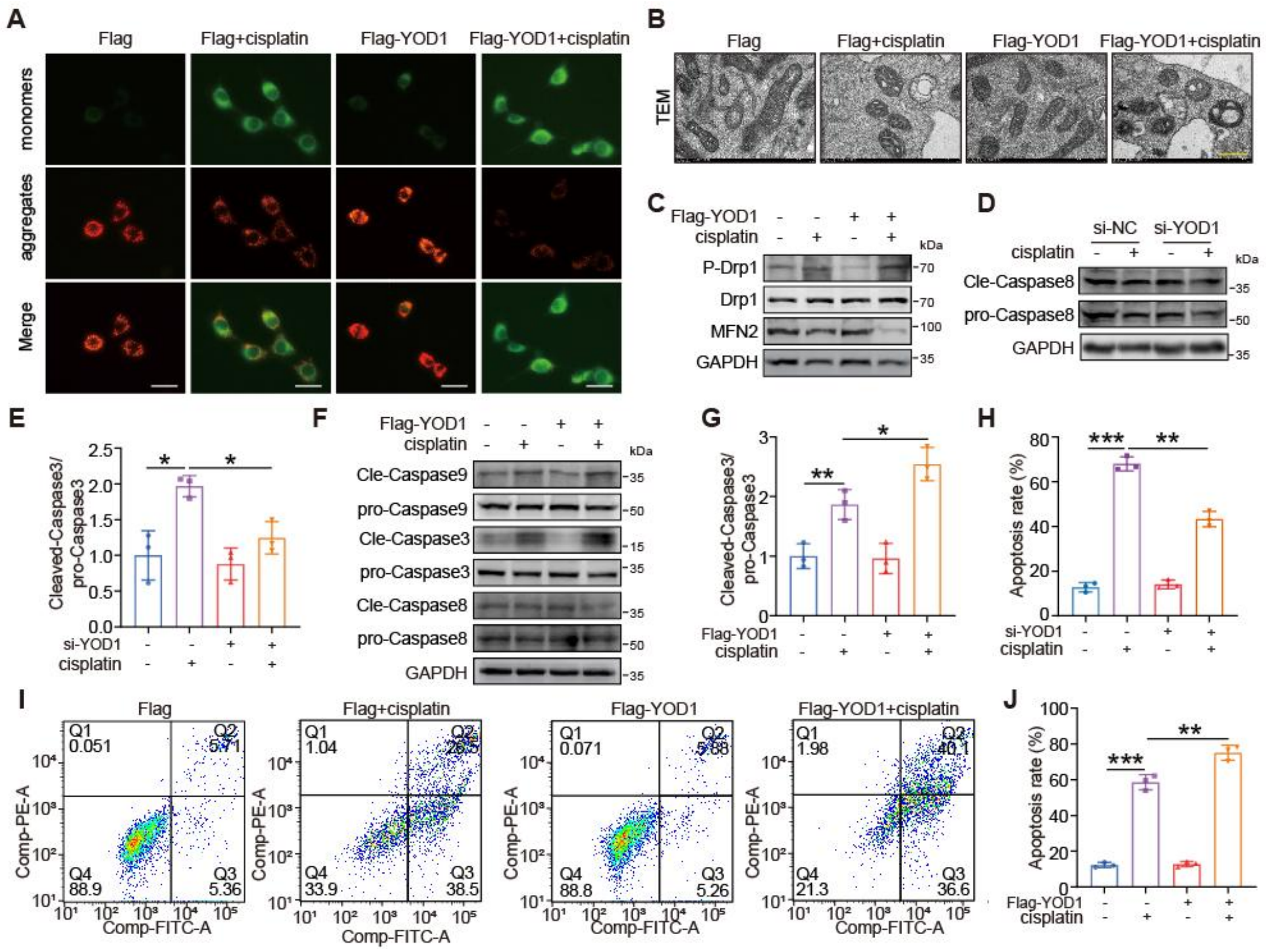

Supplementary Figure 6

(A) After transfection with Flag-YOD1, TCMK1 cells were exposed to cisplatin for 12 hours. Images of JC-1 staining on the mitochondrial transmembrane. (B-C) After transfection with Flag-YOD1, TCMK1 cells were exposed to cisplatin for 24 hours. The TEM images showing mitochondrial ultrastructure in indicated groups. Scale bar, 500 nm. Representative western blot images showing protein levels of p-Drp1 and MFN2 (n = 3). (D) After transfection with si-YOD1, TCMK1 cells were exposed to cisplatin for 24 hours. Western blot analysis showing Cleaved-Caspase8 expression levels (n = 3). (E) Quantitative densitometry of the immunoblots in Figure 6K (n = 3). (F-G) After transfection with Flag-YOD1, TCMK1 cells were exposed to cisplatin for 24 hours. Western blot analysis showing protein levels of Cleaved-Caspase9, Cleaved-Caspase3 and Cleaved-Caspase8 (n = 3). (H) Quantitative analysis of total apoptosis (sum of early and late apoptotic cells) in Figure 6L (n = 3). (I-J) After transfection with Flag-YOD1, TCMK1 cells were exposed to cisplatin for 24 hours. Cell apoptosis was analyzed by Annexin V/PI and quantitative analysis of total apoptosis (sum of early and late apoptotic cells) (n = 3).

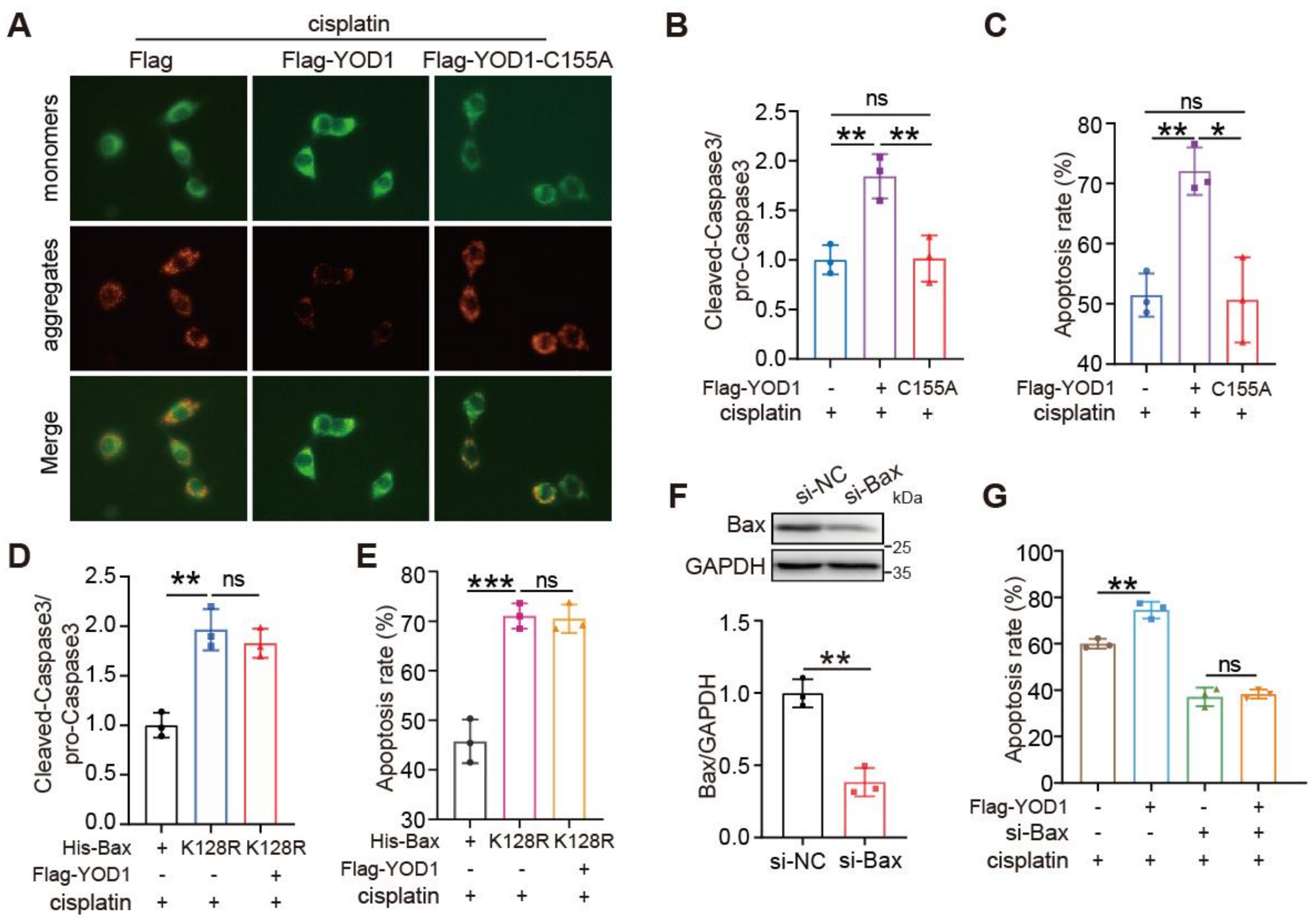

Supplementary Figure 7

(A) Flag-YOD1 (WT or C155A) was transfected into TCMK1 cells and then exposed to cisplatin for 12 hours. Images of JC-1 staining on the mitochondrial transmembrane. (B) Densitometric quantification of the immunoblots in Figure 7B (n = 3). (C) Quantitative analysis of total apoptosis (sum of early and late apoptotic cells) in Figure 7C (n = 3). (D) Densitometric quantification of the immunoblots in Figure 7E. (n = 3). (E) Quantitative analysis of total apoptosis (sum of early and late apoptotic cells) in Figure 7F (n = 3). (F) TCMK1 cells were transfected with si-Bax for 24 h, while the control cells were transfected with negative control (NC) siRNA. Representative immunoblot of Bax level (n = 3). (G) Quantitative analysis of total apoptosis (sum of early and late apoptotic cells) in Figure I (n = 3).
